# Supplementary material for: Factors contributing to neonatal mortality in a tertiary center in south West Bank: A single-center retrospective cohort study
Source: PLOS Glob Public Health. 2025 Jul 23;5(7):e0004885. doi: 10.1371/journal.pgph.0004885 (PMC12286372; doi:10.1371/journal.pgph.0004885)
Supplement: Attachment — Submitted filename: rebuttal letter.docx [file pgph.0004885.s002.docx]

- Please provide a complete Data Availability Statement in the submission form, ensuring you include all necessary access information or a reason for why you are unable to make your data freely accessible. If your research concerns only data provided within your submission, please write "All data are in the manuscript and/or supporting information files" as your Data Availability Statement.

\# done

- Please insert an Ethics Statement at the beginning of your Methods section, under a subheading 'Ethics Statement'.

#done

- Tables should not be uploaded as individual files. Please remove these files and include the Tables in your manuscript file as editable, cell-based objects. For more information about how to format tables, see our guidelines: <https://journals.plos.org/globalpublichealth/s/tables>

#done

- We have noticed that you have cited Table 4 and 5 in the manuscript file but there are no corresponding tables in the manuscript. Please amend your manuscript to include this table, noting that tables should not be uploaded as individual files.

#done

- Reviewer #1: There is no Table 4 or Table 5 to review. Please attach for proper review of entire manuscript and results. This should be a minor revision just to attach the tables.

\# done

- Recommend copy editing for appropriate spelling, grammar, and use of abbreviates (e.g., lines 30-34).
  # done

- Lines 48-49: this is a strong point and can speak to the lack of literature on Palestinian health in general. It may be worthwhile to mentioned studies that have focused on neonatal mortality in the region or in Palestinian refugee populations. For example, <https://doi.org/10.1016/S0140-6736(13)60206-8> or <https://doi.org/10.1016/S0140-6736(17)32061-5>
  # done
- Recommend rewording Line 54

\# done

- Explicitly state any exclusion criteria in the study design and sample section, and the rationale. For example, why were neonates who were admitted to NICU for less than 24 hours not included

\# done

- The methods used in the Data Analysis section were a little unclear. The use of chi-square tests and then multiple regressions could be more clearly stated with variables of interest clearly delineated. For example, why is preeclampsia and subsequent ESRD especially noted? Also, bivariate logistic regression was previously stated in the manuscript but not clearly defined (i.e., what are the two binary dependent variables?). Sometimes odds ratio is reported while other times adjusted odds ratio. Perhaps this would be clearer with Tables 4 and 5, but those were not attached to the manuscript.

\# done

- Line 99: what qualifies a mother to be listed as “healthy”? Is this a self-report variable or gleaned from the electronic health record?

\# done

- For Tables 1-3: there seems to be information from the mother and neonate dispersed throughout the tables. Consider organizing based on those two categories, where summary statistics on maternal characteristics are distinguished from patient admission characteristics.

\# done

- Line 106: consider revising for more clear interpretation of the odds ratio.

\# done

- Line 136: consider removing personal tense “our”.
  # done
- Line 152-153: consider if mentioned if those therapies are available in Palestine, and the overall resource scarcity that may complicate treatment of neonates.

\# done

- Line 154: consider removing phrasing “trend towards significance” as it not sound in the study of statistics. Consider reporting effect size for stronger analysis to assess the magnitude of difference, if data is available. A minimal suggested revision would be to change the phrasing

\# done
